# Role of Enzyme Technologies and Applied Enzymology in Valorising Seaweed Bioproducts

**DOI:** 10.3390/md23080303

**Published:** 2025-07-29

**Authors:** Blessing Mabate, Lithalethu Mkabayi, Deandra Rochelle Goddard, Coleen Elizabeth Grobler, Brett Ivan Pletschke

**Affiliations:** Enzyme Science Programme (ESP), Department of Biochemistry, Microbiology and Bioinformatics, Rhodes University, Makhanda 6139, South Africa; bmabate@gmail.com (B.M.); lithalethum@gmail.com (L.M.); deandragoddard@gmail.com (D.R.G.); coleen1521@gmail.com (C.E.G.)

**Keywords:** bioproducts, biorefinery, circular bioeconomy, enzyme-assisted, seaweeds

## Abstract

Seaweeds, classified as non-vascular plants, have definite advantages over terrestrial plants as they grow rapidly, can be cultivated in coastal environments, and are dependable and non-endangered sources of biomass. Algal bioproducts, which include a wide range of bioactive compounds, have drawn much interest because of their applications in nutraceuticals, pharmaceuticals, agriculture, and cosmetics. Particularly in the pharmaceutical and nutraceutical fields, algal bioproducts have shown tremendous activity in regulating enzymes involved in human diseases. However, the drawbacks of conventional extraction methods impede the complete exploitation of seaweed biomass. These include low efficiency, high cost, and potential harm to the environment. Enzyme technology developments in recent years present a viable way to overcome these challenges. Enzymatic processes improve product yields and reduce the environmental impact of processing, while facilitating the more effective extraction of valuable bioactive compounds as part of an integrated biorefinery approach. Enzyme-assisted biorefinery techniques can greatly advance the creation of a circular bioeconomy and increase the yield of extracted seaweed bioproducts, thus improving their value. With the potential to scale up to industrial levels, these biotechnological developments in enzymatic extraction are developing rapidly and can advance the sustainable exploitation of seaweed resources. This review emphasises the increasing importance of enzyme technologies in the seaweed biorefinery and their contribution to developing more environmentally friendly, economically feasible, and sustainable methods for valorising products derived from seaweed. In the biorefinery industry, enzyme-assisted methods have enormous potential for large-scale industrial applications with further development, opening the door to a more sustainable, circular bioeconomy.

## 1. Introduction

Seaweeds, also known as marine macroalgae, are significant photosynthetic organisms in coastal ecosystems. Seaweeds are globally recognised for their diverse applications in various industries and their essential ecological roles. These productive marine macrophytes contribute to climate change mitigation by sequestering carbon dioxide through photosynthesis. As primary producers, they decrease greenhouse gases, generate oxygen, and enhance the stability of the marine ecosystem [1]. Seaweed farming has emerged as a crucial sector of the economy, providing coastal communities worldwide with sustainable means of subsistence [2]. The rich nutritional profile of seaweed, which includes proteins and bioactive compounds that fight a variety of health issues, is driving up its demand in foods and nutraceuticals worldwide; thus, the global seaweed market has grown significantly over the last 20 years, tripling in size [3]. In response to environmental concerns about plastic waste, innovations in seaweed-based biodegradable materials are becoming more popular as environmentally friendly substitutes for traditional plastics [4].

Moreover, seaweed products have shown a myriad of promising medically relevant bioactivities, particularly by targeting enzymes involved in the pathophysiology of human diseases such as Type 2 Diabetes Mellitus (T2DM), obesity, and Alzheimer’s. Seaweed polysaccharides and phlorotannins are compounds that lower postprandial blood glucose levels by inhibiting enzymes such as α-amylase and α-glucosidase. According to research studies, seaweed extracts can reduce fat absorption by blocking pancreatic lipase activity [5]. In addition, bioactive seaweed peptides may control blood pressure by naturally inhibiting the angiotensin-converting enzyme (ACE) [6]. Marine algae metabolites, such as phenolics and alkaloids, have also shown neuroprotective effects in preclinical models, suggesting new treatment options for neurodegenerative illnesses [7]. This review will highlight that seaweed-derived bioactive compounds have therapeutic potential in disease prevention and management, despite the current difficulties in producing these compounds on an industrial scale.

Seaweed valorisation faces several obstacles that prevent it from reaching its full potential, especially in bioproduct extraction. The inefficiencies of the current extraction techniques, the underutilisation of leftover biomass, and the requirement for sustainable and profitable processes are the main reasons for these challenges. For instance, the industrial use of valuable bio-compounds such as polyphenols, alginates, and fucoidan is limited by the low yields obtained from conventional extraction methods [8]. Single compound extraction may undervalue the algal biomass by leaving behind valuable components in the waste. To improve yield and sustainability, new techniques are being developed, such as sequential and enzyme-assisted extraction [8]. These methods aim to maximise the recovery of bioactive compounds under mild, environmentally friendly conditions. In line with the tenets of the circular bioeconomy, the biorefinery approach presents a viable means of making the most of seaweed biomass. However, to fully realise the potential of seaweed valorisation, issues with scalability and economic viability continue to be major obstacles that must be overcome. To highlight the potential of seaweed bioproducts in addressing global issues such as food security, health management, and sustainable development, this review aims to thoroughly evaluate their diverse applications in important industries, such as nutraceuticals, pharmacology, agriculture, and cosmetics. Further exploring the critical role of enzymology in the extraction, refinement, and value addition to bioactives derived from seaweed, the review highlights the role of creating integrated biorefinery approaches. The field of bioproduct extraction is changing due to the specificity and environmental friendliness of enzyme-assisted extraction (EAE) procedures, which increase yields and maintain bioactivity while reducing environmental impact. The cost, stability, and scalability of enzymes are still issues which restrict their wider industrial use. To get past these obstacles, developments in enzyme engineering, immobilisation methods, and process optimisation are being investigated. This will bring enzymatic technology in line with the circular bioeconomy and sustainability principles. By discussing these topics, the review hopes to address knowledge gaps and offer insights for future uses of seaweed bioproducts to develop more environmentally friendly and sustainable industries.

## 2. Background to Seaweeds

Seaweeds are macroscopic, multicellular marine algae that use sunlight as energy to produce carbohydrates from carbon dioxide and water [9]. They are categorised according to their size, pigments, ecology, habitat, morphology, and composition of polysaccharides. Seaweeds are further divided into three groups: brown seaweed (Phaeophyta) has an estimated 1800 species, green seaweed (Chlorophyta) has about 6700 species [10], and red seaweed (Rhodophyta) has 6500 species [11]. Cold waters are ideal for seaweed growth. They are primarily found in the oceans’ intertidal and upper sublittoral zones, though they can grow as deep as 30 to 50 m. Medium-wavelength green light is typically absorbed by seaweeds for photosynthesis. Naturally occurring in coastal regions, they have a very high productivity rate. According to estimates, the productivity of brown algae that are not cultivated can range from 3.3 to 11.3 kg dry weight m^−2^ per year in terms of dry weight [9].

Seaweeds are frequently subjected to unfavourable environmental factors, such as UV light and high reactive oxygen species (ROS), causing oxidative stress. To counteract this, seaweeds generate antioxidants like carotenoids and polyphenols, which counteract ROS and shield cellular constituents from harm [12]. Seaweeds respond to environmental stressors such as temperature fluctuations, salinity, and UV radiation by synthesising bioactive compounds. Alginates from brown seaweeds, carrageenans from red seaweeds and ulvans from green seaweeds are examples of bioactive substances that seaweeds produce as cell wall structures [13]. To support their survival and ecological roles, these polysaccharides preserve cell integrity, offer protection from environmental stressors, and promote interactions with other organisms [13]. Seaweeds are also exposed to various microorganisms in the marine environment; thus, they create bioactive substances with antimicrobial qualities to protect against possible pathogens, which lowers biofouling and prevents dangerous microbial colonisation [14]. Therefore, seaweeds produce a wide range of metabolites for their survival, including lipids, polyphenols, and various carbohydrates.

### 2.1. Structures of Seaweeds

**Figure 1:**
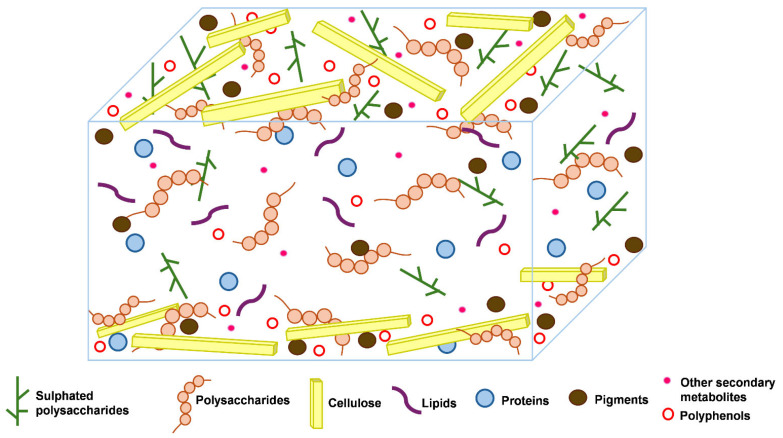
General structure of seaweed cell walls. Modified from [16]. Although different types of seaweeds exist, this structure depicts the components within the cell walls, and specifics are described in the text.

Seaweed cell walls are complex structures that differ greatly between the three groups of algae: green (Chlorophyceae), red (Rhodophyceae), and brown (Phaeophyceae). They differ mainly in the composition of polysaccharides, which are tailored to their unique physiological and ecological requirements. The general structure of seaweeds consists of a microfibrillar network embedded in a matrix of various proteins and polysaccharides that make up their cell wall (Figure 1). This composition offers protection from environmental stressors, mechanical strength, and flexibility [15]. Brown algae cell walls are characterised by extensive amounts of alginates [8] and fucose-containing sulphated polysaccharides (fucoidans), which dominate in quantity over cellulose [16] and laminarin. In addition to these major polysaccharides, Salmeán and colleagues [17] also reported the presence of β-glucans with a mixed linkage of β-(1,3)-*D*-glucans, and β-(1,4)-*D*-glucans, deemed to be structural elements that resemble plant callose. Red algae, on the other hand, are characterised by sulphated polysaccharides, including agar (comprising agarose and agaropectin) with distinct gel-like properties, and carrageenan provides red algae cell wall rigidity and flexibility [18]. Due to the complexity of their cell walls, red algae also contain cellulose, xylan, or mannan fibrils, along with many matrix polysaccharides. The cell walls of green algae contain sulphated glucuronoxylorhamnans, mainly ulvans [18]. Although sulphated polysaccharides are present in green algae, they are less abundant than in brown and red algae. Additionally, the composition of their cell walls is simpler [19].

Even though these different classes of algae exhibit these distinct characteristics of cell wall components, they generally also contain a variety of sulphated polysaccharides and cellulose, which produce microfibrils that reinforce the cell wall structure (Figure 1). All these classes of seaweeds also contain varying amounts of proteins and polyphenols (Figure 1), which are present in relatively low quantities compared to the polysaccharides. Understanding the exact composition and structure of seaweed cell walls is essential for several applications, such as the effective extraction and development of bioproducts and their investigation in pharmaceutical, agricultural, nutraceutical, and cosmetic products. Therefore, ongoing research is required to fully elucidate the structure of seaweeds.

### 2.2. Applications of Seaweeds and Their Bioproducts

Seaweeds and their derivatives have been used for various purposes in many cultures, such as for food, medicine, and agricultural inputs. Seaweeds are used in various dishes, including salads and condiments, and are still an essential part of the Asian diet [20,21]. In addition, traditional knowledge has documented the use of seaweeds as medicine in home remedies. They were and are still applied freshly, dried, or as mucilage extracts to treat burns, rashes, and scurvy as well as to help get rid of parasites [22]. Also, seaweeds have long been used as fertilisers to improve soil fertility, especially in Europe [23]. In ancient times, this method was essential to the agricultural output of coastal communities. Furthermore, kelp ash—which is made from burning seaweed—has historically been important for producing alkali and iodine. Furthermore, kelp-derived alginate, a carbohydrate, has been used as a thickening agent in toothpaste, ice cream, jelly, and salad dressings [24]. These historical applications highlight the versatility and significance of seaweeds in a range of cultural, medicinal, agricultural, and industrial contexts.

To date, with the advancement of science, and bioproduct extraction techniques in particular, including enzyme-assisted approaches, these historical seaweed applications can be amplified for the improved valorisation of seaweed bioproducts. In this regard, we have outlined the major algal value-added products and their applications according to their classes (Table 1). Bioproducts from brown algae include alginate, fucoidan, and polyphenols. The diverse bioactive compounds found in brown seaweeds, such as fucoidan, alginate, and polyphenols, have made these bioproducts popular. For example, alginate, the main polysaccharide found in brown algae, has been used in the nutraceutical industry as a stabilising and thickening agent in food products. Also, because alginates can form hydrogels and are non-toxic, they are widely used in the biomedical field in tissue engineering, drug delivery systems, and wound dressings [25]. In agriculture, alginates improve water retention and nutrient delivery by acting as seed coatings and soil conditioners [26].

**Table 1:** Bioproducts from unique classes of seaweeds.

Seaweed Type	Sector	Bioproducts	Applications	References
Brown	Pharmaceutical	Fucoidan, polyphenols/phlorotannins	Antidiabetes, anticancer, antioxidant, antiviral	[27,28,29]
	Nutraceutical	Fucoxanthin & alginates	Antioxidants, weight management & immune boosters	[25]
	Agricultural	Alginates, growth hormones	Biostimulants & soil conditioners	[26]
	Cosmetic	Alginates & phlorotannins	Anti-ageing creams, moisturisers & sunscreens	[30]
Red	Pharmaceutical	Carrageenan & agar	Drug delivery systems, anticoagulants & antiviral agents	[31,32]
	Nutraceutical	Agar & protein extracts	Dietary fibre, prebiotics & functional foods	[33]
	Agricultural	Carrageenan & whole extracts	Plant growth promoters	[34]
Green	Pharmaceutical	Ulvan, polyphenols	Antioxidants, immunomodulatory, & antibacterial agents	[35,36]
	Nutraceutical	Polyphenols and protein extracts	Functional foods, anti-inflammatory supplements	[37]
	Agricultural	Whole extracts	Biofertilisers, plant defense enhancers	[38]

Brown seaweed also produces fucoidan, a sulphated polysaccharide that has attracted much interest due to its many uses and potential for commercialisation. Fucoidan exhibits biological activities, including antioxidant, anti-inflammatory, and wound-healing activities [29]. In addition, fucoidans have demonstrated remarkable antidiabetic properties by inhibiting amylolytic digestive enzymes [27,39] as well as anticancer properties where they inhibited adhesion, migration and long-term survival of human colorectal cancer cells [28]. These have the potential to be marketed as substances with commercial value in pharmaceuticals and nutraceuticals. Other bioactive brown seaweed products include polyphenols, particularly phlorotannins. Phlorotannins have anti-inflammatory, antidiabetic, anticancer, and antioxidant qualities [40]. In addition, they have demonstrated potential as chemopreventive agents, controlling apoptotic pathways and lowering oxidative stress—two functions that are essential in cancer treatment [41]. In addition, studies suggest that phlorotannins may have antiviral properties, including blocking of virus entry and inhibition of proteins that are required for viral replication, which could lead to potential uses in the treatment of viral infections [42]. The marketing of dietary supplements and cosmetics enriched with these compounds has increased because of a growing awareness of their health benefits [30]. The increasing commercial interest and potential for innovation have led to phlorotannin-containing products being patented [41]. Phlorotannins have a lot of potential uses, but there are still concerns regarding their stability and bioavailability. Thus, more research is necessary to optimise their potential across multiple industries and enhance their commercial feasibility.

Red seaweeds (Rhodophyta) are composed of more than 50% polysaccharides (carrageenan and agar), which are widely used as gelling, thickening, and stabilising agents in the food, cosmetic, and pharmaceutical industries [31]. Furthermore, red seaweeds are high in protein and provide essential amino acids that are safe for human consumption [33]. They also contain antioxidative, anti-inflammatory, and neuroprotective pigments, such as phycoerythrin and essential fatty acids [43]. Examples of emerging applications include animal feed, bioethanol production, and functional foods. Compounds derived from red seaweed also exhibit promise in biomedicine, such as anticoagulant, antimutagenic, anticancer, and antidiabetic properties [32]. *Pyropia* and *Gracilaria* are two economically important agarophyte and carrageenanophyte species that are grown worldwide for these products [44].

Green seaweeds also contain a unique polysaccharide known as ulvan, which exhibits tremendous bioactivities, including antibacterial, anti-inflammatory, and anticancer potencies, making them valuable for therapeutic applications [35,36]. Green seaweeds are a great plant-based substitute for animal protein because they have high protein content and are a source of essential amino acids [37]. Antioxidants and vitamins found in these seaweeds help fight oxidative stress. Due to their nutritional qualities, green seaweeds have also been utilised in the creation of functional foods like enriched bread and gluten-free pasta [45]. Also, green seaweed bioproducts have attracted interest in the agricultural industry, mainly as fertilisers and biostimulants. These products support sustainable agricultural practices by boosting crop resilience, nutrient uptake, and general plant health [38].

## 3. Marine Bioproducts Inhibiting Enzymes Involved in Human Diseases

Among their many therapeutic potentials, these bioactive compounds have been shown to target key enzymes implicated in various diseases. This section outlines the known bioactivities of algal bioproducts against several human metabolic diseases through specific enzymatic pathways (Table 2). Diabetes Mellitus (type 1 or type 2) is characterised by high blood sugar (hyperglycaemia) due to defective insulin regulation [46]. Most of the research has focussed on Type 2 Diabetes Mellitus (T2DM), the most common form, stimulated by hyperglycaemia, causing insufficient insulin secretion or insulin resistance. Digestive enzymes, α-amylase and α-glucosidases are vital in breaking down complex carbohydrates into glucose. Thus, inhibiting these amylolytic enzymes is a plausible approach to reducing postprandial hyperglycaemia [27]. Incretin hormones, including glucose-dependent insulinotropic polypeptide (GIP) and glucagon-like peptide-1 (GLP-1), stimulate insulin secretion in a glucose-dependent manner. They are rapidly degraded by dipeptidyl-peptidase-4 (DPP-IV), limiting their ability to lower blood glucose [47]. Incretins also promote pancreatic β-cell proliferation and inhibit apoptosis, increasing β-cell mass and insulin production. Pharmacological strategies for T2DM include enhancing GIP and GLP-1 secretion and inhibiting DPP-IV [47]. DPP-IV inhibitors reduce glucagon levels, delay gastric emptying, and lower blood glucose, making them a key focus in antidiabetic research [48]. Thus, seaweed bioproducts have been shown to inhibit DPP-IV, adding to their value. Protein tyrosine phosphatase 1B (PTP1B) is an enzyme that acts as a negative regulator of insulin by dephosphorylating the insulin receptor (IR) along with its substrate (IRS1) in the insulin signalling pathway. When PTP1B is overexpressed, protein tyrosine kinase (PTK) activity is reduced, causing insulin resistance by preventing insulin and its receptor from interacting. PTP1B binds to and dephosphorylates tyrosine kinase downstream of the Janus-Activated Kinase 2 (JAK2) leptin receptor in the leptin pathway. By dephosphorylating JAK2 and the signal transducer and activator of transcription 3 (STAT3), PTP1B inactivates these signalling molecules, preventing the leptin receptor from responding to leptin, contributing to leptin resistance [49]. Thus, the enzyme is a prospective therapeutic strategy for T2DM [50].

We should also mention a few studies that have been established to showcase the efficacy of seaweed bioproducts to date. Zonarol, a hydroquinone extracted from *Dictyopteris polypodioides* [51], exhibited the highest inhibition of α-amylase and α-glucosidase, with IC_50_ values of 19.29 mg/L and 6.03 mg/L, respectively. Zonarol was found to be a mixed inhibitor for α-amylase and a competitive inhibitor for α-glucosidase, the same form of inhibition as acarbose, a commercial inhibitor. The study confirmed the inhibition of increasing blood glucose levels by testing in diabetic mice; the zonarol at a dosage of 100 mg/kg (9.12 mmol/L) had a more significant effect than acarbose (8.2 mmol/L) (*p* > 0.05). Fucoidan, a sulphated polysaccharide, has also shown impressive inhibition of α-glucosidase (IC_50_ = 19 μg/mL) and synergistic efficacy when combined with acarbose for controlling postprandial hyperglycaemia [52]. This study also highlighted the potential of using many algal bioproducts in complementary medicine. In addition, a few studies have also identified seaweed bioproducts as inhibitors of DPP-IV. Edible Irish brown seaweed extracts, *Alaria esculenta* and water extracts of *Laminaria digitata*, were found to be potent inhibitors of the enzyme with almost 100% inhibition [48]. Few studies within the scope of seaweed products have investigated DPP-IV inhibition; this can probably be attributed to the high cost of the enzyme. Another enzyme that has been targeted using seaweed bioproducts is PTP1B. A study by Dhara and Chakraborty [50] extracted and characterised new 2-furanone analogues from *Turbinaria ornata*. The turbinafuranone B analogue (IC_50_ = 2.42 mM) showed more significant inhibition of PTP1B than the sodium metavanadate (IC_50_ = 2.52 mM) standard [50]. Structure-bioactivity analysis revealed that the higher topological polar surface area and optimal hydrophobicity of turbinafuranone B facilitated its interaction with the active sites of the PTP1B enzyme, as confirmed by the favourable docking parameters observed (binding energy of −11.80 kcal/mol and docking score of −12.78 kcal/mol) [50]. These findings highlight the potential of turbinafuranone B, a marine-derived cyclooctafuranone, as a promising candidate for anti-hyperglycaemic treatment.

Obesity is a chronic disorder characterised by excessive weight gain due to increased fat deposition, resulting from a lower energy expenditure than caloric intake [53]. Managing obesity is crucial to reducing the risk of chronic diseases and mortality. One approach involves inhibiting pancreatic lipase enzymes that participate in fat metabolism. These inhibitors diminish fat absorption, consequently reducing lipid levels [53]. Since obesity and diabetes are closely related, the α-amylase, α-glucosidase, DPP-IV and PTP1B enzymes are also involved in anti-obesity activity. Another enzyme important in controlling obesity is the angiotensin-converting enzyme (ACE). ACE converts angiotensin I to angiotensin II, which acts through AT1 and AT2 receptors—AT2 promotes fat storage, while AT1 inhibits fat breakdown [54]. One study generated peptide hydrolysates, polyphenol and polysaccharide extracts from *Phyllospora comosa*, *Ecklonia radiata*, and *Ulva ohnoi*, and tested their bioactivities. The peptide hydrolysates extract of *U. ohnoi* had the highest inhibition of ACE (IC_50_ = 167.52 ± 3.17 μg/mL). The *P. comosa* (polyphenol) and *E. radiata* (peptide hydrolysates) extracts had IC_50_ values for pancreatic lipase ranging from 52.14 ± 2.77 μg/mL to 876.30 ± 34.92 μg/mL, respectively [55]. A murine study tested if supplementing *Gloiopeltis furcata* polysaccharides into a high-fat diet would have an anti-obesity and anti-diabetic effect. Interestingly, the mice treated with *G. furcata* polysaccharides had reduced weight gain and fat accumulation in the liver and the body, with increased levels of glucose and cholesterol in the blood combined with increased faecal excretion of fat compared to mice only fed a high-fat diet. The study suggested that inhibition of pancreatic lipase was the mechanism of action [56].

Other significant metabolic human diseases are mentioned in Table 2, which shows the efficacy of seaweed bioproducts against enzymes involved in neurodegenerative diseases, including Alzheimer’s and Parkinson’s. Also, seaweed products have been reported to inhibit enzymes involved in inflammation and virus proliferation (Table 2). In this review, we elected to focus on T2DM and obesity-associated enzymes (α-amylase, α-glucosidase, and pancreatic lipase) at the site where algal compounds are in the alimentary canal, with no complications in molecular transport to the site of action. With this brief outline, we demonstrate the huge potential and value of seaweed products in combating some human metabolic diseases.

Although seaweeds are rich in bioactive substances such as polysaccharides, polyphenols, and vital minerals, they have enormous potential as a sustainable resource for a range of uses, including pharmaceuticals, biomaterials, and nutraceuticals; extracting these efficiently still poses challenges to date. The limitations of established traditional extraction methods -which frequently involve harsh chemicals, high energy inputs, and drawn-out processes that may degrade sensitive bioactive compounds or result in low yields—hinder the full exploitation of this potential [27]. These disadvantages underscore the pressing necessity for novel and effective methods, like enzyme-assisted extraction (EAE), which provides a more environmentally friendly, focused, and sustainable substitute. By utilising enzymatic specificity, this method maximises the bioavailability of the intended bioproducts while minimising the environmental impact, thereby releasing the full potential of seaweeds in contemporary applications.

**Table 2:** Seaweed bioproducts with inhibitory effects on enzymes involved in human diseases.

Seaweed Bioproduct	Source (Seaweed Type)	Target Enzyme	Disease Association	Mechanism of Action	Biological Effect	Reference
Fucoidan	*Ecklonia radiata*	α-Amylase, α-glucosidase	Type 2 Diabetes, Obesity	Inhibits carbohydrate hydrolysis, reducing postprandial glucose levels	α-Glucosidase: IC_50_ 19 μg/mL.	[52]
	*Sargassum binderi*	α-Glucosidase, pancreatic lipase	Type 2 Diabetes, Obesity	Prevents fat digestion and glucose absorption	α-Glucosidase (IC_50_ = 174.63 ± 23.94 μg/mL)	[57]
	*Padina tetrastromatica*	3C-like protease (3CL^pro^),	Coronavirus	Disrupts coronavirus replication and blocks viral entry, collectively hindering infection and propagation	3CL^pro^: IC_50_ 0.37 mg/mL	[58]
Laminarin	*Laminaria digitata* and *Fucus vesiculosus*	Cyclooxygenase-1 (COX-1) and cyclooxygenase-2 (COX-2)	Inflammation	Preventing tissue damage, modulating immune responses, and alleviating inflammatory conditions	Crude: IC_50_ = 48.96 µg/mL (COX-1) and IC_50_ = 42.74 µg/mL (COX-2)	[59]
	*Laminaria digitata* and *Fucus vesiculosus*	Dipeptidyl Peptidase-IV (DPP-IV)	Type 2 Diabetes	Enhances insulin secretion by inhibiting DPP-IV	Crude laminarin: 46.84% inhibition at 1 mg/mL	[59]
Carrageenan Oligosaccharides	*Eucheuma cottonii*	α-Amylase	Type 2 Diabetes	Lowers glucose absorption	*α*-amylase: 59.33% inhibition at 1000 ppm	[60]
Ulvan	*Ulva lactuca*	Angiotensin-converting enzyme (ACE)	Obesity and Hypertension	Reduce vasoconstriction and lower blood pressure and prevent fat breakdown and absorption for obesity	Ulvan oligosaccharides: 50.18% inhibition	[61]
	*Ulva* species	α-Glucosidase	Type 2 Diabetes	Lowers glucose absorption	Completely hydrolysed: IC_50_ = 2.51 ± 0.19 mg/mL	
Polyphenols	*Phyllospora comosa*	α-Amylase, ACE, and pancreatic lipase	Diabetes, Obesity and Hypertension	Lowers glucose absorption. Reduces vasoconstriction and lowers blood pressure and prevents fat breakdown and absorption for obesity	ACE: IC_50_ = 583.76 ± 9.42 α-Amylase: IC_50_ = 58.31 ± 1.41 μg/mL Pancreatic lipase: IC_50_ = 52.14 ± 2.77 μg/mL	[55]
Bioactive Peptides (Hydrolyzed seaweed proteins)	*Phyllospora comosa* and *Ulva ohnoi*	α-Amylase, ACE, and pancreatic lipase	Diabetes, Obesity and Hypertension	Lowers glucose absorption. Reduces vasoconstriction, lowers blood pressure and prevents fat breakdown and absorption for obesity	ACE: IC_50_ = 167.52 ± 3.17 μg/mL (*U. ohnoi*) α-Amylase: IC_50_ = 423.39 ± 18.60 μg/mL (*U. ohnoi*) Pancreatic lipase: IC_50_ = 742.48 ± 30.37 μg/mL (*P. comosa*)	[55]
Sterols	*Sargassum horridum*	Acetylcholinesterase (AChE)	Alzheimer’s Disease	Prevents acetylcholine breakdown, improving cognitive function	Potent non-competitive inhibition of AChE	[62]

## 4. Enzyme-Assisted Extractions (EAEs)

Unlike the conventional chemical method, which solely relies on solvents, enzyme-assisted extraction (EAE) utilises an enzyme’s ability to disrupt algal cell walls, thereby releasing bioactive compounds from the seaweed [27]. Some commonly used commercial enzyme preparations include Viscozyme^®^ L, Celluclast^®^ 1.5 L, Flavourzyme^®^, amylase, protease, glucanase, Termamyl^®^, Ultraflo^®^, Alcalase^®^, agarase, xylanase, amyloglucosidase, Neutrase^®^, Kojizyme^TM^, and Protamex^®^ [30,63]. The choice of enzyme depends on the target compounds and the type of seaweed. This is mainly because bioactive compounds, such as seaweed polysaccharides, are generally tightly associated with cellulose, hemicellulose, and proteins, creating a chemically complex and heterogeneous structure [16]. Therefore, the disruption of this complex structure of cellulose, hemicellulose, and protein by cellulases, hemicellulases, and proteases leads to the release of the bioactive compounds of interest.

Optimisation of extraction parameters, such as temperature, pH of the system, solvent type (water or buffer), particle size of the substrate, agitation rate, extraction period, enzyme concentration, and enzyme selection, is critical for maximising the efficiency of the EAE technique and obtaining targeted bioactive compounds with the desired properties [8,63]. Furthermore, studies have shown that combining different enzymes that synergistically hydrolyse seaweed can enhance the efficiency of releasing the bioactive compounds [8]. This is because most of the desired compounds are embedded within the complex algal cell wall structure, and their release often requires enzymes with different specific activities to break down various components of the cell wall. In addition, EAE can be utilised alone or in combination with other eco-friendly novel techniques such as microwave-assisted extraction (MAE) and ultrasound-assisted extraction (UAE) to maximise the valorisation of seaweed biomass.

Several studies have reported comparisons of EAE and conventional extraction methods for obtaining bioactive compounds from seaweed. Olivares-Molina and Fernández [64] compared a conventional method (maceration) and an enzymatic method (cellulase and α-amylase) to prepare extracts from *Lessonia nigrescens*, *Macrocystis pyrifera*, and *Durvillaea antarctica*, and to evaluate them as inhibitors of the angiotensin I-converting enzyme (ACE). The results showed that the employment of α-amylase on *L. nigrescens* provided the highest extraction yield (37.72%) and the highest ACE inhibition (95.61%), thus demonstrating the efficiency of EAE [64]. Choulot et al. [65] compared EAE with conventional water extraction to recover plant growth regulators from red seaweed *Solieria chordalis.* The authors found that EAE increased the yields compared to water extraction in terms of neutral sugars, proteins, and plant growth regulators [65]. Coextraction of phenolics and polysaccharides from the brown seaweed *Padina gymnospora* was investigated with different enzymes (Cellulast^®^, Pectinex^®^, and Alcalase^®^), individually and in combination [66]. The results showed that yields from Alcalase-assisted extraction were higher than those obtained using either water alone or conventional ethanol extraction. Furthermore, radical scavenging activities of the extracts were higher than those obtained from conventional extraction methods, demonstrating that bioactive polysaccharides and phenolics can be obtained together in high yield through an efficient Alcalase-assisted extraction [66].

Rhein-Knudsen et al. [67] investigated the enzymatic extraction (cellulases and alginate lyases) of fucoidan from two brown seaweeds, *Saccharina latissima*, and *Alaria esculenta*, and compared this to a traditional dilute acid fucoidan extraction. It was found that the enzymatically extracted fucoidans contained high amounts of fucose and sulphate, the key components of fucoidans, and very low contents of coextracted polysaccharides [67]. Mabate et al. [27] compared fucoidan extraction yields from *Sargassum elegans* using EAE and two well-established extraction techniques. The results indicated improved fucoidan yield (4-fold) compared to the conventional extraction methods using water and mineral acid. In addition, it was shown that the extraction technologies yielded structurally and biochemically unique fucoidan fractions [27]. Overall, EAE can be utilised in the efficient extraction of a wide variety of seaweed bioproducts such as polysaccharides, plant-grown regulators, proteins, and phenolic compounds (Table 3). These reports show that this technique can provide significant benefits such as increasing the yield, improving product purity, and preserving the functionalities and bioactivities of the bioproducts. Despite all the advantages, a notable limitation of enzymatic methods is their typically slow hydrolytic activities, often necessitating extended processing durations that can exceed 24 h [68]. Some reports on the EAE of different bioactive compounds from seaweeds, and their associated bioactivities, are summarised in Table 3.

**Table 3:** Seaweed bioproducts from enzyme assisted extractions and their bioactivities.

Seaweed Species	Bioactive Compound	Extraction Conditions	Enzyme	Yield (Dry Weight)	Bioactivity	Reference
*Kjellmaniella crassifolia*	Fucoidan	50 mM citrate buffer (pH 4.8) at 50 °C for 10 h	Cellulase and β-glucosidase (ratio: 1:4.29)	4.74%	Antioxidant activity	[69]
*Cystoseira myrica*	Fucoidan	Digestion with papain for 24 h at 60 °C	Papain (100 mg)	3.07%	Antioxidant and anticoagulant activities	[70]
*Padina arborescens*	Fucoidan	Deionized water, pH (4.5) at 50 °C, shaking for 24 h	Celluclast (1%, mg/g of biomass)	26%	Anti-inflammatory	[71]
*Fucus vesiculosus*	Plant growth regulators, neutral and reducing sugars and phenolics	Deionized water, enzymes 5% (enzyme/seaweed ratio (*w*/*w*)) at 50 °C, shaking for 17 h	β-glucosidase, β-1,3-glucanase, Botrytis glucanase, and protease (neutral, endo)	Reducing sugars (21%), neutral sugars (10%)	Antioxidant activity	[72]
*Gracilaria dura*	Protein extracts	Citrate buffer solution (0.1 M, pH 4.5), shaking at 35 °C for 20 h	Hemicellulase	212.57 mg/g (protein/extract)	Emulsifying and antioxidant activities	[73]
*Porphyra dioica*	Peptides	Deionized water, pH (8.0) at 50 °C, shaking for 120 min	Prolyve 1000^®^ and Flavourzyme^®^	Protein content (25.65%)	Antioxidant activity	[74]
*Ulva rigida*	Ulvan	Deionized water, pH (8.0) at 50 for 4 h	Alcalase	Not specified	Food preservative	[75]
*Ulva fenestrata*	Ulvan	0.1 M sodium acetate buffer (pH 5.0) and 50 °C (Viscozyme L) or 40 °C (Cellulysin), 0.1 M Tris HCl buffer (pH 7.0) and 60 °C (Neutrase) and pH 5.0 at 50 °C (Flavourzyme) for 20 h	Viscozyme L, Cellulysin, Neutrase and Flavourzyme	14.1% with Cellulysin	Not specified	[76]
*Fucus vesiculosus*	Fucoxanthin	0.1 M Sodium acetate buffer (pH 4.5), 50 °C, shaking for 12 h	Viscozyme	0.657 mg/g	Not specified	[77]
*Turbinaria conoides* and *Padina tetrastromatica*	Phenolic compounds	Viscozyme in acetate buffer (pH 4.5), flavourzyme in phosphate buffer (pH 7.0), alcalase in phosphate buffer (pH 8.0), 50 °C for 20 h	Carbohydrases and proteases	73.3% yield and 3.15 mg gallic acid equivalents/g extract	Antioxidant activities	[78]

## 5. Challenges in the Enzyme-Assisted Extractions of Seaweed Bioproducts

Although macroalgae harbour many desirable compounds, they are often only produced in low concentrations or are entrapped within the complex seaweed structures [63]. EAE uses enzymes to digest major structural components of the seaweed cell wall, releasing the compounds of interest into the solution. Generally, for EAE to be successful, knowledge of enzymes and the structure of biomass matrix is essential. Furthermore, enzyme extraction requires long processing times, often over 24 h. The enzymes used in EAE are also often expensive, and downstream processes are required to attempt to recover and reuse these biocatalysts [79]. Another limitation of enzyme use in compound extraction in macroalgae is substrate specificity. Most enzymes used in extraction are cellulases, which were designed for use in terrestrial plant extractions [68]. These may not always be effective as the macroalgal structure is different and complex. Although effective, enzymes can have unintended drawbacks, such as degrading the targeted compound. We have previously observed that Celluclast^®^ 1.5 L possibly cleaves fucoidan’s sulphated chains, which are important for its bioactivity [27]. The use of enzymes also has the drawback of being highly dependent on process parameters, such as temperature, pH, ionic strength, or buffer composition, which emphasises the intrinsic sensitivity and limited stability of the enzymes under varying operating conditions [68]. Despite these challenges, enzyme-based extractions present substantial potential. Areas such as enzyme discovery and engineering are crucial for improving the economic viability and sustainability of seaweed biorefineries. The field of enzyme and microbial technology has advanced in recent years, and this advancement can reduce some EAE challenges and make the process more efficient. A few advancements have taken place in metagenomics, recombinant gene technologies, and enzyme immobilisation.

## 6. Advancements Mitigating Enzyme-Assisted Challenges

### 6.1. Metagenomics

As valuable as macroalgae are in their natural form, this review has also shown how important seaweed-derived products may be derived or extracted. Although many of these products can be accessed using various extraction techniques, EAE can be precise and efficient, and obtain high-purity compounds. Metagenomics take advantage of enzymes naturally produced by microorganisms found on algal surfaces, as the bacteria and fungi which utilise parts of their host as nutrients to carry out their biochemical processes [80]. While microbial communities benefit from this interaction, so do the algae, as associated microorganisms can influence the host’s morphological development, enhance nutrient and mineral availability, and offer protection against pathogens [81].

Considering the diversity of the chemical and physical properties of these macroalgae, a single microorganism may possess various enzymes responsible for metabolising compounds within the seaweed they occupy. Furthermore, the composition of the microbial community and species found on the same type of seaweed from different locations may differ significantly [80]. Therefore, thousands of enzymes with unique properties or metabolic pathways are still being discovered. In the past, novel enzymes were characterised using a culture-based approach, which requires finding a suitable growth medium and incubation period for the microorganism that produces these enzymes, often making it time-consuming and costly [82]. Some microorganisms only grow in very specific conditions, which cannot be reproduced, and therefore, the enzymes of interest cannot be studied using this approach [82].

Metagenomics is a technique developed to study microorganisms and their products without growing them. This approach involves extracting and enriching all bacterial and fungal genes from the selected environment, constructing a metagenome library using polymerase chain reaction (PCR) and recombinant technology techniques, screening the library, and finally, sequencing the metagenomic DNA from the library [83]. Metagenomics can access the genetic material of all the microbes associated with a particular seaweed species, providing us with insight into the biochemical reactions between the microorganism and the macroalgae [81]. This technology allows for the discovery of novel enzymes that may have commercial importance, such as high hydrolytic activity to produce seaweed-derived oligosaccharides to improve bioactivities or monosaccharides for biofuel industries.

Synthetic biology opens the doors for designing and producing new biological systems or organisms with properties tailored to our needs or optimising and overexpressing pre-existing pathways to enhance the process of interest [84]. Microorganisms or enzymes can be engineered using synthetic biology to aid in the conversion of seaweed biomass into more valuable products, ultimately increasing the extent of valorisation of seaweed-derived products [81,84]. For example, *Pseudomonas aeruginosa* and *Azotobacter vinelandii* are two alginate-producing bacterial species. The DNA responsible for alginate production by these bacterial strains was optimised and transformed into *Pseudomonas fluorescens*, which showed enhanced alginate production [84]. The optimisation of microbial alginate production is a promising avenue, as traditional methods of polysaccharide extraction can become labour-intensive [81]. Combining enhanced polysaccharide production by microbes and EAE from seaweeds using novel enzymes can allow us to unlock the full potential of seaweed valorisation.

### 6.2. Enzyme Engineering

Enzyme technology is an invaluable tool used in numerous industries to harvest novel, bulk, and improved products [85]. Wild-type enzymes typically have a narrow thermal and pH range, are solvent-specific, or are easily hydrolysed by protein-cleaving enzymes. Furthermore, the extraction or production of some of these enzymes is costly, or the enzymes are unable to function without expensive co-substrates [85]. Fortunately, with recent advances in enzyme technology, wild-type enzymes can now be engineered to suit the needs of the desired commercial application.

Marine microorganisms naturally produce hydrolytic enzymes, as these organisms cannot utilise the polysaccharides in their structurally complex form [86]. For example, alginate is cleaved by some alginate lyases to produce alginate oligosaccharides and further hydrolysed into monosaccharides by others, depending on whether they are exotype, endotype, or bifunctional lyases [87]. The enzymes produced by these marine microorganisms generally display low activity, producing a low yield of oligosaccharides [88]. The wild-type microorganisms may also not be culturable in artificial conditions. Commercially available alginate lyases are costly, often display low activity, and are limited by their physical properties [89]. Recombinant technology and genetic engineering have allowed researchers to overcome these limitations and produce alginate lyases and other similar enzymes with improved activity and physical and chemical properties [88]. This technology has also allowed enzymes to be heterologously expressed—most often in *Escherichia coli* as this host organism is easily transformed, grows quickly and cost-effectively, and expresses many recombinant proteins in high yields [90].

Recombinant expression requires isolating the gene encoding region of the enzyme and inserting this gene into the genetic material of the host organism. This has allowed for the efficient expression of various novel wild-type enzymes. For example, an alginate lyase gene *(algL*-5) naturally expressed by *Flammeovirga* sp. Strain MYO4, was codon-optimised for expression in *E. coli* to produce a new gene: *al2.* Codon-optimisation enhances the efficiency of protein translation by the host despite the enzyme being non-native [91]. In addition, the *al2* gene was modified to contain no signal peptide, further increasing expression levels and, hence, the yield of the enzyme [91]. Genetic engineering uses site-specific mutagenesis and can be used to alter undesirable characteristics. For example, enzymes used in an industrial setting must be thermostable to ensure their activity remains high throughout the reaction, which may need to run for extended periods. However, many wild-type enzymes lack thermostability [92]. Site-specific changes can be made to the enzyme’s genetic material to improve this undesirable trait. This was achieved when disulphide bonds were included in the genetic material of an alginate lyase from *Microbulbifer* sp. *Q7*, increasing its half-life time from 1.16 h to 2.25 h and increasing its activity 1.5-fold when running the reaction at 45 °C [92]. These techniques are invaluable in improving the desired characteristics of the enzymes of interest, for example, temperature, pH stability, and specificity, which improve efficiency. These technologies can significantly reduce the costs and challenges associated with the EAEs of seaweed bioproducts.

### 6.3. Enzyme Immobilisation

Constantly having to express enzymes at an industrial scale is not a financially feasible option, and it is time-consuming. Enzyme immobilisation is a technique that involves fixing the enzyme to a solid support material, maintaining and stabilising the structure of the enzyme and slowing the rate at which the enzyme is degraded by its environment [93]. This technique has allowed enzymes to display improved thermal stability and remain active despite extreme pH levels and the presence of organic solvents [94]. The support matrix is generally a porous material, such as cellulose, silica, agarose, or a range of different polymers. The support material selected will affect how stable, active and reusable the immobilised enzyme is [93]. In enzyme immobilisation, the enzyme and its support material can be recovered and reused, thereby reducing the total costs and time required for the industrial process. Enzymes can be immobilised using physical or chemical methods. To physically immobilise enzymes, encapsulation and entrapment techniques must be carried out, while the chemical mechanism involves covalently binding the enzyme and support material [93]. Covalent and/or non-covalent affinity tags can be attached using enzyme engineering, permitting the interactions between the enzymes and the support material [93]. Despite this method enabling immobilisation, the enzymes still lose activity over time due to the interaction between the surface material and the protein [93]. However, with improved genetic modification techniques, the function of immobilised enzymes can be improved through modifications that prevent these interactions.

### 6.4. Dual Valorisation Impact of Immobilised Enzymes and Seaweed Bioproducts

Not only is there the potential for immobilised enzymes to be used for the cost-effective production of seaweed-derived products, but also the potential for the use of seaweed-derived products as excellent support materials for immobilising enzymes in other industrial applications. Seaweed-derived products are naturally derived biopolymers which have gained increasing attention due to their renewability, non-toxicity, diversity, biocompatibility and range of reactive sites, which allows for a broad range of uses [94]. Biopolymers from seaweeds, including alginate, agar, and carrageenan, have recently been used as surface materials and showed excellent results, with the enzymes remaining active and stable [94].

For example, carrageenan, a large, sulphated polysaccharide derived from red seaweeds, has shown great potential for its application as a support matrix for biomolecules, whole cells, and enzymes [94]. Glucoamylase is an industrially significant enzyme capable of the hydrolysis of starch into glucose monomers through the cleavage of the glycosidic bonds found within starch. Currently, this enzyme is produced by microorganisms, such as *Aspergillus niger*, at an industrial scale in large bioreactors. However, this process requires downstream processing as removing the enzyme from the reaction is challenging [95]. The seaweed-derived biopolymer, κ-carrageenan, provided a potential solution for this. Glucoamylase was immobilised onto the polysaccharide, enhancing its pH and temperature stability and increasing its reusability, allowing it to maintain activity for 11 cycles [95]. Reusability is a very important property at an industrial scale, as it decreases production costs and reduces downstream processing times. Not only do enzymes valorise seaweed bioproducts, but the bioproducts can also valorise enzymes and improve their efficiency even beyond seaweed bioproduct processing.

## 7. Circular Bioeconomy

The circular bioeconomy is an ecosystem-driven economy that emphasises the use of renewable natural capital and waste reduction to replace the wide range of non-renewable, fossil-based products currently in use with sustainable and resource-efficient operations. In terms of seaweeds, this bioeconomy highlights how the value of seaweeds is increased through biorefinery processes, which support sustainability and the preservation of ocean ecosystems while generating renewable resources [96].

Since the announcement of the United Nations’ 17 Sustainable Development Goals (SDGs), bioeconomy initiatives have drawn special attention [96]. Furthermore, a relatively new trend in industries, biorefineries maximise the use of bioresources to produce fine and bioactive chemicals, attempting to meet zero waste technology goals and, consequently, sustainability and the circular bioeconomy [97]. This is consistent with Gunter Pauli’s concept of the “blue economy”, which refers to the sustainable use of the marine environment for economic growth and improved living standards while preserving the health of the marine ecosystem.

In recent years, significant advancements in downstream processing, including extraction and purification techniques, have been made to decrease biowastes and the use of large volumes of organic extraction solvents, as in classical traditional methods. This has led to the development of several contemporary extraction techniques, including EAE, supercritical fluid extraction (SFE), and accelerated solvent extraction (ASE) [98]. The biorefinery approach applies to marine organisms, including marine macroalgae [99]. To fully recover their bioactive metabolites and optimise extraction, macroalgae valorisation still requires further research. Nowadays, the development of environmentally friendly extraction processes is preferred over conventional extraction protocols, in addition to the pursuit of extraction technique efficiency through the extraction of bio-compounds with the highest extraction yield and associated bioactivity. These discoveries are strongly encouraged in order to adhere to green chemistry guidelines regarding extraction. Biorefinery concepts based on using natural biomass such as seaweed to produce diverse products are being developed to meet green technology requirements while reducing industrial waste to a minimum [8]. To truly create sustainable biorefineries, the circular bioeconomy considers a wide range of factors, such as waste valorisation, energy balance, life cycle assessment (LCA), and supportive policy frameworks. Although the enzymatic extraction of high-value compounds is the main focus of our study, we recognise that a thorough systems-level analysis is necessary. Our goal is to highlight the potential of enzyme-assisted extraction as a green, resource-efficient approach by placing it within the framework of circular valorisation. For a comprehensive evaluation of the viability and sustainability of seaweed-based biorefineries, future research combining LCA metrics and regulatory analysis will be essential.

### Enzyme-Assisted Biorefineries for Seaweeds

EAE has become one of the new technologies developed and applied to seaweed bioproduct extraction protocols. EAE has been proven to produce high-yield [27] and high-purity products compared to conventional extractions [67]. To mention a few (among the many studies employing EAE technology), polysaccharides and phenolics were coextracted from brown algae *Padima gymnospora* [66]. Protamex^®^ and Neutrase^®^ were used to extract plant growth biostimulants from the red algae *Soliera chordalis* [100]. Also, EAE for seaweed multiproduct extractions have been employed to obtain products like alginate, laminarin, and fucoidan. Clearly, EAE technologies are fast-growing, and thus, here we review a flexible proposed integrated biorefinery enzyme-assisted approach [8] and show how this framework is important for attaining a circular bioeconomy while observing the advocated green extraction technologies.

This study shows a flexible approach to using a biorefinery framework to extract polyphenols, calcium alginate, sodium alginate, and fucoidan. Algal biomass is defatted, polyphenols are extracted using solid-liquid extraction with aqueous ethanol, and carbohydrates, alginate, and fucoidan are separated using enzyme combinations (Cellic^®^ Ctec 2 and Viscozyme L). Yields of 10% polyphenols, 35% alginate, and 18% fucoidan were obtained using this method [8]. This process extracted about 63% of the initial seaweed biomass. The remaining residue can still be used as soil conditioners in agriculture. This approach significantly reduces the amount of waste compared to targeted compound extraction processes. Since this approach is flexible, it is possible to quantify lipids and pigments from the defatting stage, which have the potential for a series of bioactivities. Also, the flow of work can be modified to also target proteins, which are valuable in nutraceutical applications. We also emphasise the flexible sequential extraction flow, which can be adapted and optimised for any seaweed or desired bioproduct. Moreover, the process uses a significant amount of ethanol. However, this ethanol used for product precipitation can be recycled and used in the next defatting stage. Furthermore, the process does not use strong organic solvents, aligning with the advocated green extraction technologies.

## 8. Concluding Remarks

Seaweed bioproducts have drawn much interest because of their numerous uses in cosmetics, pharmaceuticals, nutraceuticals, and agriculture. Proteins, polyphenols, polysaccharides (fucoidan, alginate, or ulvan), and other bioactive substances derived from marine sources are promising for drug discovery because of their anti-diabetic, anti-inflammatory, anti-cancer, anti-microbial, and antioxidant properties. Despite major challenges such as limited clinical data and gaps in our understanding of their pharmacokinetics, toxicity, and potential drug interactions, seaweed-derived compounds have found success in other sectors. For instance, biostimulants and fertilisers made from seaweed are enhancing soil health and crop resilience, promoting sustainable agriculture. Similarly, algal extracts are widely used in cosmetics for their anti-ageing, UV-protective, and moisturising properties.

Notwithstanding these benefits, issues with extraction efficiency, adherence to green technology principles, and the widely used targeted extractions prevent algal bioproducts from being fully valorised. Conventional extraction techniques frequently utilise harsh chemicals, which degrade bioactive compounds and raise environmental concerns. Greener and nonconventional techniques such as accelerated solvent extractions, supercritical fluid extractions, ultrasonic, microwave-assisted, and enzyme-assisted extractions have recently emerged to answer problems of conventional organic solvent extractions. However, effective as they are, there are still limitations of expense, making scaling up challenging.

Moreover, biorefinery strategies that combine green technologies and enzyme-assisted extraction (EAE) have been suggested as an alternative to mitigate these challenges. Furthermore, the principles of the circular bioeconomy are addressed by this integrated biorefinery strategy of sequential enzyme-assisted approaches to promote the sustainable use of all biomass components, minimising waste and optimising resource efficiency. However, there are still drawbacks to enzyme technology, including exorbitant expenses, problems with enzyme stability, and restrictions regarding substrate specificity. Enzyme immobilisation, genetic engineering for improved enzyme function, and computational modelling for process optimisation are recent developments addressing these gaps. Targeted extractions are being refined by combining multi-enzyme systems with omics-driven methodologies, opening the door for industrial scalability. Future studies should explore creating cost-effective biorefinery models, refining enzyme formulations and investigating algal metabolite synergies for new bioactivities. A multidisciplinary strategy integrating enzyme biotechnology, marine bioproduct chemistry, and process engineering will be essential to fully utilise algal bioproducts for sustainable uses in industry, agriculture, and medicine.

## Data Availability

Not applicable.

## Abbreviations

Accelerated solvent extraction	ASE
Angiotensin-converting enzyme	ACE
Cyclooxygenase	COX
Dipeptidyl-peptidase-4	DPP-IV
Enzyme-assisted extraction	EAE
Microwave-assisted extraction	MAE
Protein tyrosine phosphatase 1B	PTP1B
Reactive oxygen species	ROS
Supercritical fluid extraction	SFE
Sustainable Development Goals	SDGs
Type 2 diabetes Mellitus	T2DM
Ultrasound-assisted extraction	UAE
